# Occult HBV infection in HCC and cirrhotic tissue of HBsAg-negative patients: a virological and clinical study

**DOI:** 10.18632/oncotarget.10909

**Published:** 2016-07-28

**Authors:** Nicola Coppola, Lorenzo Onorato, Valentina Iodice, Mario Starace, Carmine Minichini, Nunzia Farella, Giulia Liorre, Pietro Filippini, Evangelista Sagnelli, Giorgio de Stefano

**Affiliations:** ^1^ Department of Mental Health and Public Medicine, Second University of Naples, Naples, Italy; ^2^ Ninth Interventional Ultrasound Unit for Infectious Diseases, Cotugno Hospital, Naples, Italy; ^3^ Infectious diseases Unit, Caserta Hospital, Caserta, Italy

**Keywords:** occult HBV infection, anti-HBc positivity, liver cirrhosis, hepatocellular carcinoma, HBsAg mutants

## Abstract

**Aim:**

To evaluate the virological and clinical characteristics of occult HBV infection (OBI) in 68 consecutive HBsAg-negative patients with biopsy-proven cirrhosis and HCC.

**Methods:**

HBV DNA was sought and sequenced in plasma, HCC tissue and non-HCC liver tissue by PCRs using primers for HBV core, surface and x regions. OBI was identified by the presence of HBV DNA in at least two different PCRs.

**Results:**

OBI was detected in HCC tissue of 13 (20%) patients and in non-HCC liver tissue of 3 of these 13. OBI was detected in HCC tissue of 54.5% of 11 anti-HBs- negative/anti-HBc-positive patients, in 29.4% of 17 anti-HBs/anti-HBc-positive and in 5% of 40 anti-HBs/anti-HBc-negative (*p* < 0.0005). The 13 patients with OBI in HCC tissue more frequently than the 55 without showed Child-B or -C cirrhosis (53.9% *vs*. 5.5%, *p* < 0.0001) and BCLC-B or -C stages (46.1% vs. 1.8%, *p* < 0.0001). The pre-S1, pre-S2 and S region sequences in HCC tissue showed amino acid (AA) substitutions (F19L, P24L, S59F, T131I, Q129H) and deletions (in positions 4,8, 17 and 86) in the S region, AA substitutions (T40S, P124K, L54P, G76A, N222T and I273L) in pre-S1 region and AA substitutions in pre-S2 region (P41H and P66L). In the 3 patients showing OBI also in non-HCC liver tissue the S, pre-S1 and pre-S2 sequencing displayed patterns of mutations different.

**Conclusions:**

The study showed a significant correlation between OBI and the severity of liver damage, several patterns of mutations in the S, pre-S1 and pre-S2 regions in HCC tissue, some at their first description.

## INTRODUCTION

Hepatocellular carcinoma (HCC) is the fifth most common cancer in men and the ninth in women, with 554,000 and 228,000 new cases per year, respectively [1]. In addition, HCC is the second most common cause of death for cancer worldwide, estimated to be responsible for nearly 750,000 deaths per year, i.e., 9% of all cancer deaths [1]. Finally, HCC is the predominant type of primary liver cancer, arising mostly in cirrhotic livers [2].

The epidemiology of HCC reflects that of the etiological agents responsible for chronic liver diseases. In developing countries, liver cancer is strongly associated with hepatitis B virus (HBV) and hepatitis C virus (HCV) chronic infection, liver cirrhosis being the morphologic substrate for HCC development in 80% of cases. In particular, HBV chronic infection accounts for approximately 50% of all cases and, virtually, of all HCC cases observed in childhood [3]. The progressive application of universal HBV vaccination of new-born babies has significantly reduced the incidence of HBV-related HCC in developed countries, a reduction strengthened by the increasing use in the last decade of drugs suppressing HBV chronic infection in nearly all patients treated. In addition, the increasing use in recent years of directly acting antiviral agents that induce HCV eradication in nearly 95% of patients with chronic hepatitis C will significantly reduce, although perhaps not fully eliminate, the risk of developing HCC [4]. At present, the epidemiology of HCC in developed countries shows a reduced impact of both HBV and HCV chronic infections and an increasing role of non-alcoholic fatty liver diseases (NAFLD) [5].

Although poorly explored at present, the large pool of individuals with occult HBV infection represents a further risk of HCC development. Occult HBV infection (OBI), defined as the presence of HBV DNA in liver tissue (regardless of its detectability in serum) of individuals testing negative for serum hepatitis B surface antigen (HBsAg) [6], is a relatively common condition in patients with circulating antibodies to the hepatitis B core antigen (anti-HBc) [7]. There is some evidence suggesting that the persistent synthesis of undetectable amounts of viral transcripts may induce an HBV-specific immune response with resulting liver necroinflammation [8, 9], but the role of OBI on the progression of liver damage remains unclear. Despite the biological, epidemiological and clinical evidence of the oncogenic potential of overt HBV infection, the role of OBI in the development of HCC is unclear [10–14]. The information on the mutations in the HBsAg region in patients with OBI and HCC are scanty and fragmentary and do not allow conclusions to be drawn on this point [15–16].

This study was performed to assess the epidemiological, virological and clinical characteristics of OBI in HBsAg-negative subjects with HCC and to investigate the relationship between HBsAg mutations and HCC.

## PATIENTS AND METHODS

### Patients

This study was planned as prospective with a progressive enrolment by the senior investigators of two participating Liver Units in Naples, southern Italy. All consecutive patients who underwent a diagnostic liver biopsy for HCC at one of the two participating liver units from June 2013 to December 2014 were enrolled. HCC was diagnosed in accordance with the EASL/EORTC criteria [17].

Each patient underwent a complete physical examination, full liver function tests and serology for HBsAg, anti-HCV and anti-HIV. HBsAg-positive and anti-HIV-positive patients were excluded from the study. The anti-HCV-positive subjects were considered as having HCV infection. A diagnosis of NAFLD and NASH (non-alcoholic steato-hepatitis) were made according to the AASLD/ACG/AGA guidelines [18]. Alcohol intake and other potential causes of liver disease were assessed. A consumption of alcohol exceeding 30g per day for females and 40g per day for males over at least the last 6 months (information corroborated by family members in uncertain cases) was considered alcohol abuse possibly speeding up the progression of the liver disease to an unfavorable outcome. By the end of the enrolment period, 68 consecutive HBsAg-negative/anti-HIV-negative patients with biopsy-proven HCC had been recruited and 12 excluded (9 because of HBsAg positivity, 2 because of anti-HIV positivity and 1 because of not availability of liver tissue).

The stage of HCC was assessed by the criteria proposed by the BCLC (Barcelona Clinic Liver Cancer) group [19].

For each patient, a non-HCC liver specimen and an HCC specimen were obtained by US-guided percutaneous liver biopsy. Fragments of nearly 3 mg were cut away from the two extremities of the liver biopsies, usually not informative for histological diagnosis, and stored at −80°C in RNAlater solution (Qiagen GmbH, Hilden, Germany) for subsequent molecular analyses. In addition, plasma samples were collected for each patient and stored at −80°C the same day the liver biopsies were performed.

All procedures followed were in accordance with the international guidelines and with the Helsinki Declaration of 1975, revised in 1983. The Ethics Committee of the Azienda Ospedaliera Universitaria of the Second University of Naples approved the study (n°349/2013). All patients signed their informed consent for liver biopsy, the collection and storage of biological samples and for the anonymous use of their data for research purposes.

### Sero-virological methods

HBV serum markers (HBsAg, anti-HBs, anti-HBc) were sought using commercial immunoenzymatic assays (Abbott Laboratories, North Chicago, IL, USA). The anti-HCV antibody was sought using a 3rd generation commercial immunoenzymatic assay (Ortho Diagnostic Systems, Neckargemund, Germany). Antibodies to HIV 1 and 2 were sought using a commercial ELISA (Abbott Lab., North Chicago, IL, USA). Liver biochemistry and routine analyses were performed by routine methods in a Cobas Modular 6,000 automated analyzer using c501 biochemistry modules (Roche Diagnostics Ltd, Rotkreuz, Switzerland).

The fragments of liver tissue cut away from the liver biopsies were homogenized by TissueLyser (Qiagen GmbH, Hilden, Germany) for 30 seconds at 30 Hz and the DNA was extracted using microspin columns (QIAamp DNA kit, Qiagen GmbH). Plasma DNA was extracted from 200μl of plasma using microspin columns (QIAamp DNA Blood kit, Qiagen GmbH). The DNA extracted from plasma and from the homogenates of HCC and non-HCC liver tissues were analyzed for the presence of HBV DNA by three polymerase chain reactions (PCRs) using sets of primers for core, surface and x regions of the HBV genome, as previously reported [7]. For each compartment (plasma, HCC and non-HCC tissue), occult B infection was established by the presence of HBV DNA in at least two different PCR assays [6]. In subjects with OBI, the HBV genotype and the mutations in the HBV pre-S/S coding region were determined by automated sequencing, as previously described [20].

Specifically, HBV genotypes were determined by phylogenetic analysis of sequences of 500 nt of the S region by a nested PCR, using in the first PCR the outer primes F-2862 forward 5′-TCACCATATTCTTGGGAAC-3′ and R-853 5′-AGGGTTTAAATGTATAACCCA-3′, and in the second PCR the inner primers F-179 5′-CTAGGACCCCTGCTCGTGTT-3′ and R-690 reverse 5′-AATGGCACTAGTAAACTGAG -3′. The PCR products were then purified using the MinElute PCR Purification Kit (Qiagen, Qiagen GmbH); the sequencing reaction was performed using the primer forward F-179 5′-CTAGGACCCCTGCTCGTGTT-3′ and reverse R-690 5′-AATGGCACTAGTAAACTGAG-3′ with the Big Dye Terminator v 1.1 Cycle Sequencing Kit (Applied Biosystems, Carlsbad, CA, USA) in an ABI 310 Genetic Analyzer (Applied Biosystems). Sequences were aligned using the BioEdit program with reference sequences for the different genotypes deposited in GenBank (accession numbers: X02763, X51970, AF090842 for genotype A; D00329, AF100309, AB033554 for genotype B; X04615, M12906, AB014381 for genotype C; X65259, M32138, X85254 for genotype D; X75657, AB032431 for genotype E; X69798, AB036910, AF223965 for genotype F; AF160501, AB064310, AF405706 for genotype G; AY090454, AY090457, AY090460 for genotype H). Genetic distances were calculated with the Kimura-2-parameter algorithm and a neighbour-joining phylogenetic tree was constructed with the Mega 6 program http://www.megasoftware.net (Center of Evolutionary Functional Genomics, Biodesign Institute, Arizona State University, USA).

The pre-S /S coding region was amplified by nested PCR, using for the first PCR the primers F-2845 5′-CCTCATTTTGCGGGTCACC-3′ and R-996 5′-TTTGACCATACTTTCCAATCAAT-3 and for the second PCR primers F-2862 5′-TCACCATATTCTTGGGAAC-3′ and R-853 5′-AGGGTTTAAATGTATAACCCA-3′. The PCR products were then purified using the MinElute PCR Purification Kit (Qiagen, Qiagen GmbH) according to the manufacturer's instructions. Sequence reactions were performed with primers F-2862 and R-853 and the following primers: F-246 5′-GTCTAGACTCGTGGTGGACTT-3′, F-3037 5′-TTGGGGTGGAGCCCTCAGGCT-3′, F-56 5′-CCTGCTGGTGGCTCCAGTTCA-3′, R-690 5′-AATGGCACTAGTAAACTGAG-3′, using the Big Dye Terminator v 1.1 Cycle Sequencing Kit (Applied Biosystems, Carlsbad, CA, USA), according to the manufacturer's instructions, and run in an ABI 310 Genetic Analyzer (Applied Biosystems). The genotype was determined using the Genotyping Tool on http://www.ncbi.nlm.nih.gov

For anti-HCV positive patients, viral RNA was extracted from 140 μl of plasma samples using a microspin column (QIAamp RNA viral kit, Qiagen GmbH). HCV RNA was quantified by performing real-time PCR in a Light cycler 1.5 (Roche Diagnostics, Branchburg, NJ, USA), as reported in a previous paper [21]; by this method, the detection limit in plasma samples is estimated at around 40 IU/mL. HCV genotypes were determined by the HCV genotype Lipa assay (Bayer, France) following the manufacturer's instructions.

### Statistical analysis

Continuous variables were summarized as mean and standard deviation, and categorical variables as absolute and relative frequencies. The differences were evaluated by Student's *t*-test for continuous variables, while categorical variables were compared by chi-square test, using exact procedures if necessary.

## RESULTS

### Characteristics of patients

The demographic, clinical, biochemical and virological data of the 68 patients enrolled are summarized in Table 1. Thirty-nine patients (57.3%) were males and the mean age was 70.2+6.24 years. All 68 patients had biopsy proven cirrhosis, 59 (85.3%) were HCV-related (38 with genotype 1) and the remaining 9 (14.7%) NASH-related. Of the 68 patients enrolled, 17 (25.0%) were anti-HBs/anti-HBc-positive, 11 (16.2%) anti-HBs-negative/anti-HBc-positive and 40 (58.8%) anti-HBc/anti-HBs-negative. Liver cirrhosis was in the Child-A stage in 58 (85.3%) patients and in the Child-B stage in 10 (14.7%). HCC was mono-focal in 38 (55.9%) patients and multifocal in 30 (44.1%); only two patients (2.9%) had portal thrombosis. It was first diagnosed at enrolment in 48 patients (70.6%) and as a relapsing HCC in the remaining 20 (29.4%), all previously treated with radiofrequency ablation; of these 20, 11 was mono-focal and 9 multifocal, one had portal thrombosis. Sixty-one (89.7%) patients presented a BCLC stage A, 6 (8.8%) a BCLC stage B and 1 (1.5%) C (Table 1).

**Table 1:** Demographic, biochemical, virological, and clinical characteristics of the patients enrolled

N° patients	68
Mean age (±SD)	70.2 (6.24)
Males, n° (%)	39 (57.3)
Alcohol abusers(> 30g/die), n° (%)	2 (2.9)
Subjects with diabetes, n° (%)	8 (11.7)
BMI (mean ± SD)	27.1 (2.8)
AST/ULN(mean ± SD)	1.68 (0.9)
ALT/ULN (mean ± SD)	1.59 (0.8)
ALP/ULN (mean ± SD)	1.17 (0.5)
Total bilirubin, mg/dl (mean ± SD)	1.53 (3.7)
PT% (mean ± SD)	84.7 (24.6)
α−fetoprotein, (mean ± SD)	173.7 (552.7)
Anti-HCV-positive patients, n° (%) HCV-RNA-positive subjects, n° (%) HCV load, IU/mL (mean ± SD) with HCV-genotype 1, n° (%) with HCV-genotype non-1, n° (%)	59 (86.8) 47 (79.7) 1.15E6 (1.78E6) 38 (64.4) 9 (15.2)
Patients with NASH, n°(%)	9 (13.2)
Child Pugh score, n° (%) of patients with A B C	58 (85.3) 10 (14.7) 0 (0)
Patients with first diagnosis of HCC, n° (%)	48 (70.6)
Patients with HCC relapse, n° (%)	20 (29.4)
Patients with a single HCC, n° (%)	38 (55.9)
Patients with multiple HCC, n° (%)	30 (44.1)
Patients with portal thrombosis, n° (%)	2 (2.9)
BCLC score, n° (%) of patients with A B C	61 (89.7) 6 (8.8) 1 (1.5)

### Correlation between OBI and the characteristics of the patients

OBI (presence of HBV DNA in at least two different PCR assays) was detected in both HCC tissue and non-HCC liver tissue of 3 (4.4%) patients and in the HCC tissue of 10 (14.7%), whereas no patient showed an OBI in plasma. The results of the 3 HBV PCRs in the 13 patients with OBI are shown in the Supplementary Table 1. In particular, OBI was detected in 6 (54.5%) of the 11 anti-HBs-negative/anti-HBc-positive patients, in 5 (29.4%) of the 17 anti-HBs/anti-HBc-positive and in 2 (5%) of the 40 anti-HBs/anti-HBc-negative (*p* < 0.0005 by Chi-square test of Pearson). Of the 3 patients with OBI in both HCC tissue and non-HCC liver tissue, two were anti-HBs-negative/anti-HBc-positive and 1 anti-HBs/anti-HBc-positive. The 13 patients with OBI in HCC tissue compared with the 55 without (Table 2) were younger (65.7+8 years *vs*. 71.2+5.3, *p* = 0.03) and more frequently showed liver cirrhosis in the Child B stage (53.9% *vs*. 5.5%, *p* < 0.0001) and a BCLC stage B or C (46.1% *vs*. 1.8%, *p* < 0.0001) (Table 2). No other significant difference was observed between these subgroups as regards the demographic, biochemical and clinical data such as unifocal/multifocal HCC, HCC first diagnosis/relapse and the presence/absence of portal thrombosis (Table 2).

**Table 2:** Demographic and initial biochemical, serological, virological and clinical data according to the presence of occult HBV infection in HCC tissue

Parameters	Patients with occult HBV infection	Patients without occult HBV infection	*p*
N° of patients	13	55	
Mean age (±SD)	65.7 (8.0)	71.2 (5.3)	0.03*
Males, n° (%)	7 (53.8)	32 (58.2)	0.78**
Alcohol abusers(> 30g/die), n° (%)	1 (7.7)	1 (1.8)	0.26**
Subjects with diabetes, n° (%)	2 (15.4)	6 (10.9)	0.97**
BMI (mean ± SD)	28.8 (3.5)	26.6 (2.4)	0.07*
AST/ULN(mean ± SD)	1.85 (0.9)	1.64 (0.9)	0.52*
ALT/ULN (mean ± SD)	1.78 (1.0)	1.56 (0.8)	0.51*
ALP/ULN (mean ± SD)	1.38 (1.0)	1.12 (0.4)	0.41*
Total bilirubin, mg/dl (mean ± SD)	1.04 (0.5)	1.63 (4.0)	0.31*
PT% (mean ± SD)	92.8 (11.6)	83.1 (26.3)	0.06*
αfetoprotein, (mean ± SD)	97.5 (170.3)	188.9 (601.1)	0.39*
Anti-HCV-positive patients, n° (%) HCV-RNA-positive subjects, n° (%) HCV load, IU/mL (mean ± SD) with HCV-genotype 1, n° (%)	11 (84.6) 9 (81.8) 1.57E6 (2.48E6) 6 (54.5)	48 (85.4) 38 (79.2) 1.07E6 (1.64E6) 32 (68.1)	0.80** 0.99** 0.98* 0.23**
Patients with NASH, n°(%)	2 (15.4)	7 (12.7)	0.80**
Anti-HBs-negative/anti-HBc-positive, n° (%) Anti-HBs /anti-HBc-positive, n° (%) Anti-HBs /anti-HBc-negative, n° (%)	6 (46.1) 5 (38.5) 2 (15.4)	5 (9.1) 12 (21.8) 38 (69.1)	Anti-HBc pos vs neg 0.001**
Child Pugh score, n° (%): A B	6 (46.1) 7 (53.9)	52 (94.5) 3 (5.5)	<0.00001**
Patients with first diagnosis of HCC, n° (%)	8 (61.5)	40 (72.7)	
Patients with HCC relapse, n° (%)	5 (38.5)	15 (27.3)	0.45**
Patients with a single HCC, n° (%)	8 (61.5)	30 (54.6)	
Patients with multiple HCC, n° (%)	5 (38.5)	25 (45.4)	0.61**
Patients with portal thrombosis, n° (%)	1 (7.7)	1 (1.8)	0.26**
BCLC score, n° (%) of patients with Score A B C	7 (53.9) 5 (38.5) 1 (7.6)	54 (98.2) 1 (1.8) 0	Score A vs. B/C <0.00001**

* Student's t-test;

** chi-square test

### HBV genotype and mutations in the S region in OBI

Of the 13 patients with OBI, 12 had HBV-genotype D3 and one genotype A2.

The sequences of pre-S/S coding regions were found in the HCC tissue of 6 of the 13 patients with OBI, the HBV loads being too low for sequencing in the untested samples, and in all 3 with OBI in the non-HCC tissue.

#### Mutations and deletions in the pre-S1, pre-S2 and S region in the HCC tissue

Of the 6 patients for whom the pre-S/S region was sequenced in the HCC tissue, 5 showed amino acid (AA) mutations or deletions in pre-S1, pre-S2 and S regions, and one no AA mutation or deletion in all regions (Table 3). More precisely, as regards the S region, 4 patients showed AA substitutions: Q129H in two patients, P24L in one and F19L, S59F and T131I in one (Table 3). Regarding the pre-S1 region, 5 patients showed AA mutations (T40S in two patients, P124K in one, L54P and G76A in one, N222T and I273L in one) and 4 showed deletions(in positions 4, 8 and 17 in 3 patients and positions 8, 17 and 86 in one) (Table 3). Finally, 4 patients showed an AA mutation in the pre-S2 region: P41H in 3 patients and P66L in one (Table 3).

**Table 3:** mutations in the pre-S1, pre-S2 and S regions in HCC and non-HCC liver tissue

Patient	HCC tissue			Non-HCC liver tissue		
	S region	Pre-S1 region	Pre-S2 region	S region	Pre-S1 region	Pre-S2 region
Patient #1	No mutations	L54P, G76A, AA deletion in positions 4,8,17	P41H	G44G/E I92T	G91A, I255T	G99E, I147T
Patient #2	P24L	N222T, I273L	P66L	PCR negative	PCR negative	PCR negative
Patient #3	F19L, S59F, T131I	P124K, AA deletion in positions 4,8,17	P41H	PCR negative	PCR negative	PCR negative
Patient #4	Q129H	T40S, AA deletion in positions 4,8,17	No mutations	Q129H	S98T, N103D, T114I, T119P, AA deletion in positions 128,129,130,131	F75S, Q184H, AA deletion in positions 19,20,21,22
Patient #5	Q129H	T40S AA deletion in positions 8,17,86	P41H	PCR negative	PCR negative	PCR negative
Patient # 6	No mutations	No mutations	No mutations	N59T, I110L	No mutations	H114T, I165L

Thus, the mutations most frequently observed in these 6 patients were AA substitutions Q129H in the S region (2 of 6 patients), T40S in the pre-S1 (2 of 6 patients) and P41H in the pre-S2 region (4 of 6 patients) and deletions in positions 8 and 17 in the pre-S1 region (4 of 6 patients), associated with a deletion in position 4 in 3 cases and in position 86 in one.

#### Mutations and deletions in the pre-S1, pre-S2 and S region in non-HCC liver tissue

The pre-S1, pre-S2 and S regions were sequenced in the non-HCC liver tissue of all 3 patients with OBI in the non-HCC tissue. The following AA substitutions and deletions were observed: a) G44E, I92T, Q129H, N59T, and I110L AA substitutions in the S region; b) AA deletions in position 128-131 and G91A, I255T, S98T, N310D, T114I, and T119P AA substitutions in the pre-S1 region; c) AA deletions in position 19-22 and G99E, I147T, F75S, Q184H, H114T and I165L AA substitutions in the pre-S2 region (Table 3).

#### Differences in mutations and deletions in the pre-S1, pre-S2 and S region between HCC and non-HCC liver tissue

The sequencing of the S, pre-S1 and pre-S2 region of the 3 patients with OBI in HCC and in non-HCC liver tissue was compared to identify differences in the mutations and deletions.

In patient n°1 the AA substitutions and deletions differed widely between HCC and non-HCC liver tissue in the pre-S1, pre-S2, and S regions. In this patient, the HCC tissue showed no mutation in the S region, AA substitutions L54P and G76A and AA deletions in positions 4, 8 and 17 in the pre-S1 region and AA substitution P41H in the pre-S2 region, whereas the non-HCC liver tissue showed the G44G/E and I92T AA substitutions in the S region, G91A and I255T AA substitutions in the pre-S1 region and AA substitutions G99E and I147T in the pre-S2 regions.

In patient n°4, apart from the AA substitution Q129H in the S region in both tissues, sequencing in HCC and non-HCC liver tissue showed differences in the AA substitutions and deletions both in the pre-S1 region (T40S and AA deletion in positions 4, 8 and 17 for HCC and S98T, N103D, T114I, T119P and the AA deletion in position 128-131 for non-HCC liver tissue) and in the pre-S2 region (no AA substitution or deletion in HCC tissue and the AA substitutions F75S and Q184H and AA deletion in position 19-22 in the non-HCC liver tissue).

In patient n°6 no mutation was observed in the pre-S1, pre-S2 and S regions in the HCC tissue, whereas in the non-HCC liver tissue the N59T and I110L AA substitutions were observed in the S region, no mutation in the pre-S1 region and the AA substitutions H114T and I165L in the pre-S2 region.

## DISCUSSION

An OBI-defining condition was observed in HCC tissue of nearly 20% of the 68 HBsAg-negative patients investigated in this study and more precisely in half of those with circulating anti-HBc alone, in nearly one-third of the anti-HBs/anti-HBc-positive and in only 5% of the anti-HBc-negative. Instead, only 3 of the 13 patients with an OBI-defining condition in HCC tissue showed OBI also in cirrhotic tissue. Taken together, these observations might support the hypothesis that OBI favours HCC development in HBsAg-negative cirrhotic patients, but the possibility that an OBI-defining condition may persist longer in HCC than in cirrhotic tissue cannot be ruled out. Moreover, although significantly younger, the subjects with OBI showed a more advanced clinical condition, expressed for liver cirrhosis by a more severe Child-Pugh score and for HCC by a worse BCLC score. The possibility that OBI may speed the progression of HCV-related liver diseases to a more severe stage has been suggested in some previous papers [22, 23, 24] that demonstrated that patients with HCV-related cirrhosis showed a higher rate of both HCC and liver-related events (development or bleeding of oesophageal varices, encephalopathy or ascites) if carrying OBI [22]. The mechanism by which OBI may enhance liver damage and induce HCC is still unclear, but a pathogenetic role of this occult infection cannot be excluded, since HBV is an oncogenic virus whose replication may induce liver cell proliferation, hepatocyte necrosis and neo-fibrogenesis [25].

The other aim of our study was to investigate the virological characteristics of occult B infection, particularly the presence of mutations in the pre-S1, pre-S2 and S regions of HBV, both in HCC and in non-HCC tissue. Some studies on HBsAg-positive Asian populations [26–28] have shown a relationship between deletions in the pre-S region and the development of HCC. These variants may have a reduced antigenicity and, by altering the immune response, may favour the replicative activity of the virus [29]. In addition, by decreasing the expression of middle and small surface proteins, the pre-S deletions may result in an intracellular accumulation of viral particles that may induce stress in the endoplasmic reticulum, oxidative DNA damage and genomic instability possibly leading to a neoplastic transformation [30]. No datum on a possible correlation between pre-S deletions in OBI and HCC development has so far been published, and the data of the present study are the first to demonstrate AA deletions in the pre-S1 region in HCC tissue. These deletions were found in positions 4, 8 and 17 in three of the 6 patients investigated and in positions 8, 17 and 86 in a fourth patient. Despite the low number of patients investigated, the data are interesting and warrant confirmation in further studies.

Also poorly investigated is the clinical value of point mutations in the pre-S1, pre-S2 and S regions in HBsAg-positive chronic hepatitis and even less in those with OBI. A case-control study [31] recently performed in Hong Kong on 74 HBsAg-positive subjects with HCC and 148 with chronic hepatitis B used as controls showed a significantly higher frequency of a AA substitutions in position 54 of the pre-S1 region in patients with HCC. In accordance with this, we found an L54P AA mutation in the pre-S1 region in the HCC liver tissue of one of the 3 patients with OBI investigated for both HCC tissue and non-HCC liver tissue.

In 2007, Pollicino and co-workers [15] identified a mutation in position 41 of the pre-S2 region in HCC tissue in 3 of 17 patients investigated, of whom two were HBsAg-positive and one showed OBI. Again as regards the pre-S2 region in OBI, we detected the mutation P41H in the HCC tissue of 3 of the 6 patients with OBI investigated in the present study.

As regards the S region in OBI, we found the Q129H mutation in two HCC tissues and in one non-neoplastic liver tissue, a mutation located within the “a” determinant of HBsAg, already described as an escape mutation [32] but never associated with hepatic carcinogenesis.

Other AA deletions or substitutions presented in Table 3 have not been discussed here only because first described in the present study. Their clinical importance, however, cannot be excluded, but further investigations are needed for a more comprehensive discussion.

In conclusion, we found a significant correlation between the presence of OBI and the severity of liver damage in the 68 HBsAg-negative patients with liver cirrhosis and hepatocellular carcinoma investigated. Different patterns of mutations in the S, pre-S1 and pre-S2 regions were observed in the HCC tissue and non-HCC liver tissue. Several AA deletions and AA substitutions were observed, some previously deemed as possibly associated with HCC development and others described for the first time in the present study. Although the small number of patients with OBI investigated in the present study and the scanty, fragmentary data reported in the literature make it difficult to draw conclusions, the data from the present study seem interesting and might constitute cornerstones for future scientific developments.

## SUPPLEMENTARY TABLE



## Abbreviations

HBV: hepatitis B virus
HCC: hepatocellular carcinoma
OBI: occult HBV infection
NAFLD: non alcoholic fatty liver diseases
HBsAg: hepatitis B surface antigen
anti-HBc: antibody to hepatitis B core antigen
BCLC: Barcelona clinic liver cancer

## References

[1] Ferlay J, Soerjomataram I, Ervik M, Dikshit R, Eser S, Mathers C GLOBOCAN Cancer Fact Sheets: liver Cancers [Internet]. GLOBOCAN 2012 v1.0, Cancer Incidence and Mortality Worldwide: IARC CancerBase No. 11.

[2] Hong T, Gow P, Fink M, Dev A, Roberts S, Nicoll A, Lubel J, Kronborg I, Arachchi N, Ryan M, Kemp W, Knight V, Farrugia H, Thursfield V, Desmond P, Thompson A, Bell S (2016). Novel population-based study finding higher than reported hepatocellular carcinoma incidence suggests an updated approach is needed. Hepatology.

[3] El-Serag HB (2012). Epidemiology of viral hepatitis and hepatocellular carcinoma. Gastroenterology.

[4] Coppola N, Pisaturo M, Zampino R, Macera M, Sagnelli C, Sagnelli E (2015). Hepatitis C virus markers in infection by hepatitis C virus: In the era of directly acting antivirals. World J Gastroenterol.

[5] Santi V, Buccione D, Di Micoli A, Fatti G, Frigerio M, Farinati F, Del Poggio P, Rapaccini G, Di Nolfo MA, Benvegnù L, Zoli M, Borzio F, Giannini EG, Caturelli E, Chiaramonte M, Bernardi M, Trevisani F (2012). The changing scenario of hepatocellular carcinoma over the last two decades in Italy. J Hepatol.

[6] Raimondo G, Allain JP, Brunetto MR, Buendia MA, Chen DS, Colombo M, Craxì A, Donato F, Ferrari C, Gaeta GB, Gerlich WH, Levrero M, Locarnini S (2008). Statements from the Taormina expert meeting on occult epatiti B virus infection. J Hepatol.

[7] Sagnelli E, Imparato M, Coppola N, Pisapia R, Sagnelli C, Messina V, Piai G, Stanzione M, Bruno M, Moggio G, Caprio N, Pasquale G, Del Vecchio Blanco C (2008). Diagnosis and clinical impact of occult hepatitis B infection in patients with biopsy provenhepatitis C: a multicenterstudy. J Med Virol.

[8] Mason AL, Xu L, Guo L, Kuhns M, Perrillo RP (1998). Molecular basis for persistent hepatitis B virus infection in the liver after clearance of serum hepatitis B surface antigen. Hepatology.

[9] Martin CM, Welge JA, Shire NJ, Shata MT, Sherman KE, Blackard JT (2009). Cytokine expression during chronic *versus* occult hepatitis B virus infection in HIV co-infected individuals. Cytokine.

[10] Coppola N, Onorato L, Pisaturo M, Macera M, Sagnelli C, Martini S, Sagnelli E (2015). Role of occult hepatitis B virus infection in chronic hepatitis C. World J Gastroenterol.

[11] Pollicino T, Squadrito G, Cerenzia G, Cacciola I, Raffa G, Craxi A, Farinati F, Missale G, Smedile A, Tiribelli C, Villa E, Raimondo G (2004). Hepatitis B virus maintains its pro-oncogenicproperties in the case of occult HBV infection. Gastroenterology.

[12] Squadrito G, Pollicino T, Cacciola I, Caccamo G, Villari D, La Masa T, Restuccia T, Cucinotta E, Scisca C, Magazzu D, Raimondo G (2006). Occult hepatitis B virus infection is associated with the development of hepatocellular carcinoma in chronic hepatitis C patients. Cancer.

[13] Obika M, Shinji T, Fujioka S, Terada R, Ryuko H, Lwin AA, Shiraha H, Koide N (2008). Hepatitis B virus DNA in liver tissue and risk for hepatocarcinogenesis in patients with hepatitis C virus-related chronic liver disease. Intervirology.

[14] Lok AS, Everhart JE, Di Bisceglie AM, Kim HY, Hussain M, Morgan TR, HALT-C Trial Group (2011). Occult and previous hepatitis B virus infection are not associated with hepatocellular carcinoma in United States patients with chronic hepatitis C. Hepatology.

[15] Pollicino T, Raffa G, Costantino L, Lisa A, Campello C, Squadrito G, Levrero M, Raimondo G (2007). Molecular and functional analysis of occult hepatitis B virus isolates from patients with hepatocellular carcinoma. Hepatology.

[16] Chen CH, Changchien CS, Lee CM, Tung WC, Hung CH, Hu TH, Wang JH, Wang JC, Lu SN (2009). A study on sequence variations in pre-S/surface, X and enhancer II/core promoter/precore regions of occult hepatitis B virus in non-B, non-C hepatocellular carcinoma patients in Taiwan. Int J Cancer.

[17] EASL-EORTC clinical practice guidelines: management of hepatocellular carcinoma (2012). European Association for the Study of the Liver, European Organisation for Research and Treatment of Cancer. J Hepatol.

[18] Chalasani N, Younossi Z, Lavine JE, Diehl AM, Brunt EM, Cusi K, Charlton M, Sanyal AJ, American Gastroenterological Association; American Association for the Study of Liver Diseases; American College of Gastroenterology (2012). The Diagnosis and Management of Non-Alcoholic Fatty Liver Disease: Practice Guideline by the American Association for the Study of Liver Diseases, American College of Gastroenterology, and the American Gastroenterological Association. Gastroenterology.

[19] Llovet JM, Brú C, Bruix J (1999). Prognosis of hepatocellular carcinoma: the BCLC staging classification. Semin Liver Dis.

[20] Coppola N, Loquercio G, Tonziello G, Azzaro R, Pisaturo M, Di Costanzo G, Starace M, Pasquale G, Cacciapuoti C, Petruzziello A (2013). HBV transmission from an occult carrier with five mutations in the major hydrophilic region of HBsAg to an immunosuppressed plasma recipient. J Clin Virol.

[21] Coppola N, Zampino R, Cirillo G, Stanzione M, Macera M, Boemio A, Grandone A, Pisaturo M, Marrone A, Adinolfi LE, Sagnlli E, Miraglia Del Giudice E (2015). TM6SF2 E167K variant is associated with severe steatosis in chronic hepatitis C, regardless of PNPLA3 polymorphism. Liver Int.

[22] Squadrito G, Cacciola I, Alibrandi A, Pollicino T, Raimondo G (2013). Impact of occult hepatitis B virus infection on the outcome of chronic hepatitis C. J Hepatol.

[23] Shetty K, Hussain M, Nei L, Reddy KR, Lok AS (2008). Prevalence and significance of occult hepatitis B in a liver transplant population with chronic hepatitis C. Liver Transpl.

[24] Wu ZF, Xu Z, Li WS, Zhang HB, Yang N, Yao XQ, Liu FK, Yang GS (2015). Impact of occult hepatitis B virus infection on outcome after resection for non-B non-C hepatocellular carcinoma. J Surg Res.

[25] Chemin I, Guillaud O, Queyron PC, Trepo C (2009). Close monitoring of serum HBV DNA levels and liver enzymes levels is most useful in the management ofpatients with occult HBV infection. J Hepatol.

[26] Coppola N, Onorato L, Minichini C, Di Caprio G, Starace M, Sagnelli C, Sagnelli E (2015). Clinical significance of hepatitis B surface antigen mutants. World J Hepatol.

[27] Liu S, Zhang H, Gu C, Yin J, He Y, Xie J, Cao G (2009). Associations between hepatitis B virus mutations and the risk of hepatocellular carcinoma: a meta-analysis. J Natl Cancer Inst.

[28] Wang C, Teng Z, Zhu Y, Zhao AZ, Sun C (2015). Associations between pre-S deletion mutation of hepatitis B virus and risk of hepatocellular carcinoma in the Asian population: a meta-analysis. Med Sci Monit.

[29] Chisari FV, Ferrari C (1995). Hepatitis B virus immunopathogenesis. Annu Rev Immunol.

[30] Wang HC, Huang W, Lai MD, Su IJ (2006). Hepatitis B virus pre-S mutants, endoplasmic reticulum stress and hepatocarcinogenesis. Cancer Sci.

[31] Zhang AY, Lai CL, Huang FY, Seto WK, Fung J, Wong DK, Yuen MF (2015). Evolutionary changes of hepatitis B virus Pre-S mutations prior to development of hepatocellular carcinoma. PLoS One.

[32] Luongo M, Critelli R, Grottola A, Gitto S, Bernabucci V, Bevini M, Vecchi C, Montagnani G, Villa E (2015). Acute hepatitis B caused by a vaccine-escape HBV strain in vaccinated subject: sequence analysis and therapeutic strategy. J Clin Virol.

