# Supplementary material for: Occult HBV infection in HCC and cirrhotic tissue of HBsAg-negative patients: a virological and clinical study
Source: Oncotarget. 2016 Jul 28;7(38):62706–14. doi: 10.18632/oncotarget.10909 (PMC5308760; doi:10.18632/oncotarget.10909)
Supplement: Supplementary file 1 [file oncotarget-07-62706-s001.docx]

|  | HCC tissue | | |
| --- | --- | --- | --- |
| Patient | X region | Core region | S region |
| #1 | negative | positive | positive |
| #2 | negative | positive | positive |
| #3 | negative | positive | positive |
| #4 | negative | positive | positive |
| #5 | negative | positive | positive |
| #6 | negative | positive | positive |
| #7 | negative | positive | positive |
| #8 | negative | positive | positive |
| #9 | negative | positive | positive |
| #10 | negative | positive | positive |
| #11 | negative | positive | positive |
| #12 | positive | positive | negative |
| #13 | positive | positive | negative |
|  | Non-HCC tissue | | |
| Patient | X region | Core region | S region |
| #1 | negative | positive | positive |
| #4 | negative | positive | negative |
| #6 | negative | positive | negative |

**Supplementary Table**: Results of the 3 HBV PCRs in the 13 patients with OBI
